# Influence of drugs on gap junctions in glioma cell lines and primary astrocytes *in vitro*

**DOI:** 10.3389/fphys.2014.00186

**Published:** 2014-05-16

**Authors:** Zahra Moinfar, Hannes Dambach, Pedro M. Faustmann

**Affiliations:** ^1^International Graduate School of Neuroscience, Ruhr University BochumBochum, Germany; ^2^Department of Neuroanatomy and Molecular Brain Research, Ruhr University BochumBochum, Germany

**Keywords:** gap junction, glioma, astrocyte, pharmaceutical preparations, microglia

## Abstract

Gap junctions (GJs) are hemichannels on cell membrane. Once they are intercellulary connected to the neighboring cells, they build a functional syncytium which allows rapid transfer of ions and molecules between cells. This characteristic makes GJs a potential modulator in proliferation, migration, and development of the cells. So far, several types of GJs are recognized on different brain cells as well as in glioma. Astrocytes, as one of the major cells that maintain neuronal homeostasis, express different types of GJs that let them communicate with neurons, oligodendrocytes, and endothelial cells of the blood brain barrier; however, the main GJ in astrocytes is connexin 43. There are different cerebral diseases in which astrocyte GJs might play a role. Several drugs have been reported to modulate gap junctional communication in the brain which can consequently have beneficial or detrimental effects on the course of treatment in certain diseases. However, the exact cellular mechanism behind those pharmaceutical efficacies on GJs is not well-understood. Accordingly, how specific drugs would affect GJs and what some consequent specific brain diseases would be are the interests of the authors of this chapter. We would focus on pharmaceutical effects on GJs on astrocytes in specific diseases where GJs could possibly play a role including: (1) migraine and a novel therapy for migraine with aura, (2) neuroautoimmune diseases and immunomodulatory drugs in the treatment of demyelinating diseases of the central nervous system such as multiple sclerosis, (3) glioma and antineoplastic and anti-inflammatory agents that are used in treating brain tumors, and (4) epilepsy and anticonvulsants that are widely used for seizures therapy. All of the above-mentioned therapeutic categories can possibly affect GJs expression of astrocytes and the role is discussed in the upcoming chapter.

## Introduction

Gap junctions (GJs) are composed of 12 subunits of connexin (Cx) in a way that each six connexins compose one connexon. The opposing connexons on the neighboring cells form a GJ through which small molecules up to 1 KD (second messengers, ATP, Ca^2+^ ions, etc.) can rapidly transfer in a network of connected cells. GJs exist in almost all cell types except mature skeletal muscles, spermatozoa, and erythrocytes (Dermietzel and Spray, 1993).

Although GJs possess some general features, they also exhibit specific characteristics depending on the subtypes, cell types and tissues. So far, 21 subtypes of Cxs have been found (Sohl and Willecke, 2003). In the brain, neurons (Cx43, Cx32, Cx36), oligodendrocytes (Cx32, Cx47, Cx29), astrocytes (Cx43, Cx30, Cx26), and microglia (Cx43, Cx36, Cx32) express different Cxs (Rouach et al., 2002; Nagy et al., 2004; Giaume and Theis, 2010); however, microglia expression of Cx43 is limited to specific brain conditions such as injury or inflammation (Eugenin et al., 2001; Giaume and Theis, 2010).

Besides the role as a channel, GJs may also exhibit hemichannel activity, which is independent of their channel permeability characteristics. Hemichannel activity of GJs refers to actions that do not require the formation of a channel between opposing connexons of the neighboring cells. It also means that their opening state depends on specific conditions in the cell milieu and according to available data and facilitates the transfer of gluthation, prostaglandin E_2_, ATP and glutamate between extracellular compartment and cytoplasm (Stout et al., 2002; Ye et al., 2003; Bruzzone et al., 2005; Cherian et al., 2005; Saez et al., 2005; Rana and Dringen, 2007).

On the other hand, hemichannel and channel activity can be differentially regulated by certain stimulus. For example, the inflammatory stimulus oppositely modulates the hemichannel and channel activity of Cx43 on both astrocytes and C6 glioma cell lines (De Vuyst et al., 2007; Retamal et al., 2007). C6 cells showed reduction of Cx43 channel permeability under FGF-2 (fibroblast growth factor-2) and LPS (lipopolysaccharide) stimulation; however, the hemichannel activity was increased. Likewise, the treatment of astrocytes with the conditioned medium of LPS-activated microglia, decreased dye coupling and gap junctional communication (GJC) in astrocytes and enhanced the hemichannel activity of Cx43 on astrocytes (Retamal et al., 2007). Hemichannel features of GJs also have major roles in cytoskeletal organization and rapid normalization of toxic levels of Ca^2+^ as well as cell proliferation, migration, adhesion, and differentiation during development. Finally, channel-dependent and channel-independent features of GJs contribute to tumor cell adhesion, migration and proliferation just like glioma (Huang et al., 1998; Lin et al., 2002, 2003; Bates et al., 2007; Cotrina et al., 2008; Decrock et al., 2009; Crespin et al., 2010).

Microglia and astrocytes are major glial cells in the brain and play important roles in maintaining homeostasis of neuronal environment (Dermietzel et al., 1991). Astrocyte dysfunction has been related to neuroautoimmune diseases, neoplasms and epilepsy (Louis, 2006; Brinkmann, 2009; De Lanerolle et al., 2010). The main focus of this chapter is astrocytes and their function in therapeutic strategies in regard to GJs and diseases. Authors will explore the effects of therapeutic agents on astrocytes' GJs in migraine, demyelinating disease of the central nervous system (CNS), glioma and epilepsy.

## Discussion

### Migraine

#### Introduction

Migraine is recognized by repeated severe pulsating unilateral headaches accompanied by photophobia, nausea and transient neurological symptoms. Migraine with aura is a category in which, headache is followed by visual disturbances. Several hypotheses have been proposed for the development of migraine with aura. A very old theory (vascular theory) proposed that the rebound vasodilation following vasoconstriction of intracranial arteries is the cause of perivascular sensory fibers and consequently pain (Pietrobon and Striessnig, 2003). However, due to lack of convincing evidence, this theory was argued and currently it is believed that some unknown molecular changes due to cortical spreading depression (CSD) generation are the cause of migraine. Neuronal excitements are thought to be the origin of CSD, that is, the spreading of a cortical wave signal to the brain cortex. CSD is believed to be the cause of several regional changes in the extracellular fluid such as increasing the concentration of K^+^ ions, nitric oxide, protons, and glutamate and thus vasodilation of blood vessel in the brain. Consequently, perivascular sensory fibers, branches of afferent trigeminal nerve, transfer the data to the trigeminal nerve ganglia; and sensitization of several pathways and nuclei in the brain stem causes pain (Olesen et al., 1990; Bolay et al., 2002; Pietrobon and Striessnig, 2003; Moskowitz, 2007; Silberstein, 2009).

Although the main cause of migraine initiation, according to the CSD, seems to be neuronal activity, the data derived from recent studies indicate an intimate role of satellite glial cells in the trigeminal nerve as a major contributing and modulating factor. Recently, it has been shown that astrocytes and their GJs might contribute to the development of migraine (Silberstein, 2006; Damodaram et al., 2009) and modulations of GJs can be helpful in migraine treatment. In this article, we are trying to address the possible importance of GJs in the treatment of migraine.

#### Tonabersat and gap junctions

Because of the physiological characteristics of GJs, they could be related as contributing factors for CSD theory. Astrocytes in the close vicinity of synaptic cleft can receive “slip over” of neurotransmitters and respond by sending Ca^2+^ wave to connected astrocytes via GJs or even send signals to remote astrocytes which are not physically connected to them by GJs (Araque et al., 1999). In either way, it was postulated that those astrocytes surrounding the ganglial neurons in trigeminal nerve have the potential to take part in CSD activity and migraine pathology (Thalakoti et al., 2007). SB-220453 (Tonabersat), with a promising anti-epileptic activity, was tested for this assumption in migraine and showed a significant positive outcome in the treatment of migraine with aura in rat and further in human (Chan et al., 1999; Damodaram et al., 2009; Silberstein, 2009).

Tonabersat was first identified as an anti-epileptic drug (AED) with specific but unknown binding sites in the brain that was different from the commonly known AEDs. In addition, it had no side effects on peripheral tissues such as heart, liver, and kidney (Herdon et al., 1997; Upton et al., 1997). Due to its effect on reducing plasma protein extravasation in rat trigeminal ganglion (Chan et al., 1999), it was studied as a potential candidate for migraine headache therapy (Parsons et al., 2001). Tonabersat affected Cx26 GJC between satellite glia cells and neurons in the sensory part of the trigeminal nerve and prevented CSD (Damodaram et al., 2009). Tonabersat reduced the neuroinflammation and inhibited CSD, which could finally reduce migraine attacks in animal models, as well as in human. Similarly, in an *in vivo* experiment, Tonabersat reduced the elevated level of Cx26 in V1 and V2 regions which was previously increased by TNF-α (an inflammatory cytokine) and capsaicin (Damodaram et al., 2009). This finding implied a significant role for GJs of astrocytes in the mechanism of action of Tonabersat in migraine therapy.

Beside the direct effect of Tonabersat on neuro-glia GJs, it exhibited an indirect influence on GJs by activating microglia *in vitro*. The microglia activation was a late response (>24 h) followed by CSD induction and it was reversible (Gehrmann et al., 1993); however, it could theoretically impose changes on the GJs expression of astrocytes and consequently their interaction with neurons and migraine. Although the functional coupling between microglia and astrocytes through Cx43 has not been confirmed, microglia modulates decrease the expression and function of astrocytic Cx43 *in vitro* by releasing cytokines (Faustmann et al., 2003; Retamal et al., 2007). As a result, we can assume that a part of Tonabersat's effect on neuro-glial GJs can be mediated through an indirect effect on activation or increased number or of regional microglia.

#### Conclusion

Tonabersat showed significant efficacy in the treatment of migraine with aura. Although the mechanism of its effect is not fully understood, the available data suggest a strong role for GJs that are connecting neurons and satellite ganglion cells in trigeminal nerve. On the other hand, its indirect effect on microglia activation can further influence the micro-milieu of neurons and consequently their firing activity. However, whether GJC inhibition is the main pharmacological mechanism of Tonabersat in human is the subject of further studies.

### Neuroautoimmune diseases

#### Introduction

Multiple sclerosis (MS) is a chronic demyelinating disease of the CNS which is characterized by degeneration of oligodendrocytes and consequently demyelination of neurons (Compston and Coles, 2008). This further causes neuronal damage and axonal loss and subsequent neurological deficits. Similarly, in neuromyelitis optica (NMO), a variant of MS, demyelination occurs but with a different pathophysiology and localization. Although the etiology of both diseases is unknown, NMO and MS are categorized separately since 2006 (Wingerchuk et al., 2006). Aquaporin4 (AQP4) is a water channel and is expressed on the end-feet of astrocytes. Recent studies show that unlike MS, circulating aberrant antibodies against AQP4 are highly raised in the sera of patients with NMO (Lennon et al., 2004; Wingerchuk et al., 2006).

#### Demyelination and gap junctions

The etiology of MS and NMO is associated with immune cells (T and B cells), although the initiating cause is still unknown and several contributing factors such as genetic predisposition, infections and vaccination, vitamin D deficiency, and environmental factors have been suggested. Few studies have addressed the role of GJs in neuroinflammatory diseases of MS or NMO (Ibrahim et al., 2001; Brand-Schieber et al., 2005; Roscoe et al., 2007a,b).

Cx43 expression was evaluated in experimental autoimmune encephalomyelitis (EAE) model of MS. For example, lumbar spinal cord of EAE showed a significant reduction of astrocytic Cx43, specifically in monocyte infiltrated areas (Brand-Schieber et al., 2005). The reduction of Cx43 can be correlated to the local release of some inflammatory properties of the lesion such as the release of pro-inflammatory cytokine of interleukin-1 (IL-1) (John et al., 1999). Interestingly, the reduction of Cx43 recovers and even exceeds the normal baseline during remyelination (Roscoe et al., 2007b). Due to lethal consequences of the deletion of Cx43 in Knockout mice, Roscoe et al. could only study remyelination in Cx43 +/− (heterozygous null mutated) or Cx43 +/+ (wild type) mice. CT301 (α4-integrin blocker) or ADAC (adenosine amine congener) improved clinical score and facilitated the remyelination of EAE guinea pigs. Despite differences in Cx43 expression in these models, disease progression was similar in both types (Roscoe et al., 2007a). On the contrary, the severity of loss of Cx43 in human brain biopsies was associated with a worse course of MS (Masaki et al., 2013). Therefore, the major question of whether de/remyelination is caused by or is a cause of Cx43 modulations, as Kielian suggested still remains unanswered (Kielian, 2008).

A number of experiments on Cx Knockout mice (Cx43, Cx30, Cx32, Cx47) showed massive demyelination in the EAE model inferring the role for connexin in demyelinating diseases such as MS (Menichella et al., 2003; Lutz et al., 2009; Magnotti et al., 2011). Masaki et al. investigated Cx expression by immunohistochemistry in 11 autopsied specimens of MS and NMO (Masaki et al., 2013). They showed more intense Cx43 and Cx30 staining in normal gray matter than in white matter, especially at foot process of astrocytes. In contrast, Cx30 level on astrocytes was very low in NMO and MS lesions. Immunoreactivity to Cx43 was completely lost in highly degenerative GFAP positive astrocytes within the active lesion of MS or NMO. On the other hand, Cx43 was up-regulated in chronic lesions. The severity of loss of Cx43 was correlated with the clinical course of NMO and MS, that is, extensive loss of Cx43 in the lesion was related to highly annual relapse rate and rapid course of the disease. Interestingly, anti-Cx43 antibody in the sera was negative in all samples (Masaki et al., 2013). In general, the differential expression of Cx43 in active and chronic lesion implies a distinguished role for Cx43 on different stages of inflammation in MS and NMO; however, the related mechanism and how exactly Cx43 contributes in this process are unknown yet.

#### FTY720 and gap junctions

MS has no cure but there are advanced therapies, including new oral therapies, preventing the progression of the disease (Gold, 2011). They mostly modulate the immune system or the attachment sites of immune cells to the endothelial layer of brain vessels. FTY720 (Fingolimod) is a new oral treatment for MS and its major function is to hold pathologic lymphocytes in the secondary lymphoid tissue in order to delay their release to the blood stream and impede further brain damage (Matloubian et al., 2004). FTY720 is a modulator of sphingosine 1-phosphate (S1P) receptor with significant efficacy in the treatment of MS patients (Brinkmann, 2009). Acting primarily on T cells, FTY720 down-regulates S1P receptor 1 (S1P1), the receptor that T cells need to express in order to escape the lymph node (Matloubian et al., 2004).

Likewise, inflammation down-regulates S1P1 and entraps T cells in the lymph node to optimize immune response in the body (Schwab and Cyster, 2007). Sphingomyelin (part of the cell membrane) degradation is the source of S1P in the body. Although all cells can produce it, platelets and erythrocytes are the major suppliers in plasma (Sano et al., 2002; Pappu et al., 2007). S1P plasma level is usually low but it will rise during inflammation which can impact various cells in which S1P receptors are expressed. Other than lymphocytes, astrocytes express S1P receptors (S1P1, S1P3) as well as oligodendrocytes and microglia/macrophages. Accordingly, S1P could play a role in astrogliosis and neurodegenerative diseases (Waeber and Chiu, 1999; Sorensen et al., 2003; Anelli et al., 2005; Jaillard et al., 2005; Kimura et al., 2007).

#### Inflammation, S1P, and gap junctions

GJ's functions are modulated by several factors including neurotransmitters and proteins. Interestingly, Rouach and colleagues evaluated the S1P effect on the GJC of astrocytes. They found that S1P has a potent inhibitory effect on GJC and electrical coupling of Cx43 of astrocytes by increasing dephosphorylated Cx43 (Rouach et al., 2006). Dephosphorylation of Cx43 protein imposes structural changes on Cx43 that finally reduces functional GJC between astrocytes. They also showed that there was no correlation between inhibition of GJC and mitotic activity. However, further *in vivo* studies were not performed to evaluate Cx43 GJC inhibition of astrocytes by S1P. As the authors suggested, S1P could have a potential role in reactive astrogliosis in brain. Due to the inflammatory nature of MS pathogenesis and possible raise of S1P either in serum or the surroundings of astrocytes, these findings implicate the role of S1P modulations of GJs on astrocytes that in turn could have further impacts on MS progression.

It is speculated that microglia, another important glia in brain, do not couple through Cx43 with astrocyte or each other, except for special situations like traumatic tissue cases (Eugenin et al., 2001). However, it can influence astrocyte coupling through diverse indirect mechanisms such as cytokine release (Faustmann et al., 2003; Hinkerohe et al., 2005; Retamal et al., 2007). For instance, interferon-beta (IFNβ) restored the reduction of astrocytes' GJC caused by pro-inflammatory cytokines (IFNγ, IL-1β, and IL-6) in cultured astrocytes (Hinkerohe et al., 2005). In addition, Cx43 expression showed a strong negative correlation with microglia phenotype. Taken together, we can conclude that IFNβ, that is widely administered for MS patients (McCormack and Scott, 2004), can contribute to neutralizing the inflammatory environment of astrocytes and GJ expression and consequently help MS treatment (Hinkerohe et al., 2005). However, the long term efficacy of such a treatment in reducing disability of MS patients has been doubted (Shirani et al., 2012).

#### Conclusion

Despite the lack of definite evidence for the role of GJs in the pathology of MS or NMO, these findings could imply the role of GJs as contributors or modifying factors during MS therapy or pathogenesis. Whether Cx43 is the cause or effect of certain inflammation like cytokine release in demyelination pathology is a subject to be investigated in further studies.

### Glioma

#### Introduction

Brain neoplasm is a rare condition (<2% frequency); however, it is lethal with poor prognosis (<1 year survival rate) (Parkin, 2001; Parkin et al., 2001). So far, only palliative treatments like surgery and chemo/radio therapy are available but none of them can cure the disease (Sin et al., 2012). There are two common theories proposed for the origin of glioma: (1) astrocytes transformed to a malignant type and (2) cancer stem cells (Singh et al., 2003; Louis, 2006; Vescovi et al., 2006). These theories are based on either the similar morphology of astrocytes to tumor cells or the migration pattern of neural crest cells and glioma cells (Dirks, 2001). The idea behind cancer stem cell as an origin for brain tumor arose from the identification of stem cells among leukemic cells. These stem cells possess the ability of proliferation and self-renewal that would make them sufficient and necessary for tumor progression and maintenance. The same pattern has been recognized in brain tumor where stem cells were isolated from different types of brain neoplasms. These cells showed similar phenotypes although they were collected from different types of tumors (Singh et al., 2003).

#### Gap junctions and glioma gene therapy

Cx43 expression is very heterogeneous in glioma; however, most of the studies indicate that it has an inverse association with glioma grade and is less expressed in glioma than normal tissue (Soroceanu et al., 2001; Pu et al., 2004). Therefore, attempts have been made to take advantage of Cx43 modulations in the treatment of glioma like gene therapy.

In gene therapy, the main purpose is to insert a gene into tumor cells that finally makes them sensitive to special medications. Herpes simplex virus-thymidine kinase (HSV-TK) suicide gene therapy has been used to treat glioma (Ram et al., 1997; Nicholas et al., 2003; Immonen et al., 2004). In HSV-TK gene therapy, ganciclovir (GCV) treatment will further kill the infected cells. However, scientists encountered a special phenomenon in this model which was then called “bystander effect.” It was noticed that the neighboring cells that were not induced by HSV-TK were also killed after ganciclovir therapy in animal experimental models and total cell deaths outnumbered the transfected cells (Moolten and Wells, 1990; Culver et al., 1992; Nicholas et al., 2003).

Several hypotheses were raised to explain the bystander effect: (1) non-infected cells died because of phagocytosing toxic metabolites of dead cells, (2) the immune system became activated against tumor cells, (3) certain toxic metabolites were transferred through cell-cell communication. Further studies suggested the last theory as a better explanation for this effect. For example, as the phosphorylated form of ganciclovir (GCV-P) cannot cross cell membrane, the presence of GCV-P in the neighboring non transfected cells was argued to be mediated by GJs (Nicholas et al., 2003). Similarly, tumor cell lines, unable to transfer calcein dye, did not show the bystander effect, either. Calcein is a dye that only passes through GJs and the inhibition of its transfer to neighboring cells infers to the inhibition of GJC. Furthermore, increasing GJC led to more bystander cell death such as the influence seen from apigenin or lovastatin in murine adenocarcinoma cells (Touraine et al., 1998a,b).

Similar effects were observed in C6 cells. Normally, C6 cells show less GJC (Naus et al., 1991); however, once they are transfected with Cx43, they show higher GJC and bystander cell death (Dilber et al., 1997; Robe et al., 2000). Surprisingly, in gene therapy studies, the efficacy of bystander cell death was mostly dependent on the Cx43 expression of the non TK-transduced cells rather than the infected cells (Nicholas et al., 2003), a phenomenon which emphasizes that higher GJC enhances the bystander effect.

Besides Cx43, the role of other Cxs was investigated in glioma gene therapy. Three different Cxs (Cx26, Cx32, Cx43) and their effects on C6 glioma cell line proliferation and HSV-TK gene therapy were further studied (Jimenez et al., 2006). Cx26 and Cx32 had the most potent role in the efficient bystander effect of HSV-TK therapy and Cx43 significantly contributed to this effect. Modulation of Cxs was not directly evaluated at protein level; however, the findings indirectly demonstrated the contribution of Cx43 to glioma cell proliferation through measuring cell survival after ganciclovir treatment (Jimenez et al., 2006). In conclusion, these findings imply that for a better clinical approach, the higher the expression of Cx43 in glioma cells is, the better the prognosis for HSV-TK treatment would be.

Along with the role of Cx43 as a channel in glioma, studies show that Cx43 can act as a tumor suppressor gene, as well (Zhu et al., 1992; Goodenough et al., 1996; Omori and Yamasaki, 1998; Huang et al., 1999; Zhang et al., 2003a). Investigating C6 cells transfected with Cx43, Zhang et al. showed that Cx43 elevated p27 (cyclin-dependent kinase inhibitor) (Zhang et al., 2001, 2003b). They also showed the decreased level of proto-oncogene SKP2 (S phase kinase-associated protein) which is probably the main cause of P27 reduction in C6 cells. The authors could also clearly demonstrate that these effects were mediated by C-terminal of Cx43, independent of channel permeability of Cx43. Therefore, Cx43, as a hemichannel, could inhibit cell growth and applying this hypothesis in the glioma therapy could prove beneficial.

#### Adjuvant chemo/radio therapies, Cx43 and glioma

To achieve increased Cx43 and consequently bystander effect, adjuvant chemo/radio therapies have been studied. Dexamethasone (DEX) is commonly used as a symptomatic therapy for glioma patients to reduce edema and inflammation (Kaal and Vecht, 2004). However, the administration of DEX is a matter of debate because it can also be in favor of tumor growth by reducing the sensitivity of tumor cells to common palliative therapies like chemo/radiotherapy (Weller et al., 1997; Gorman et al., 2000; Das et al., 2004).

**Figure 1:**
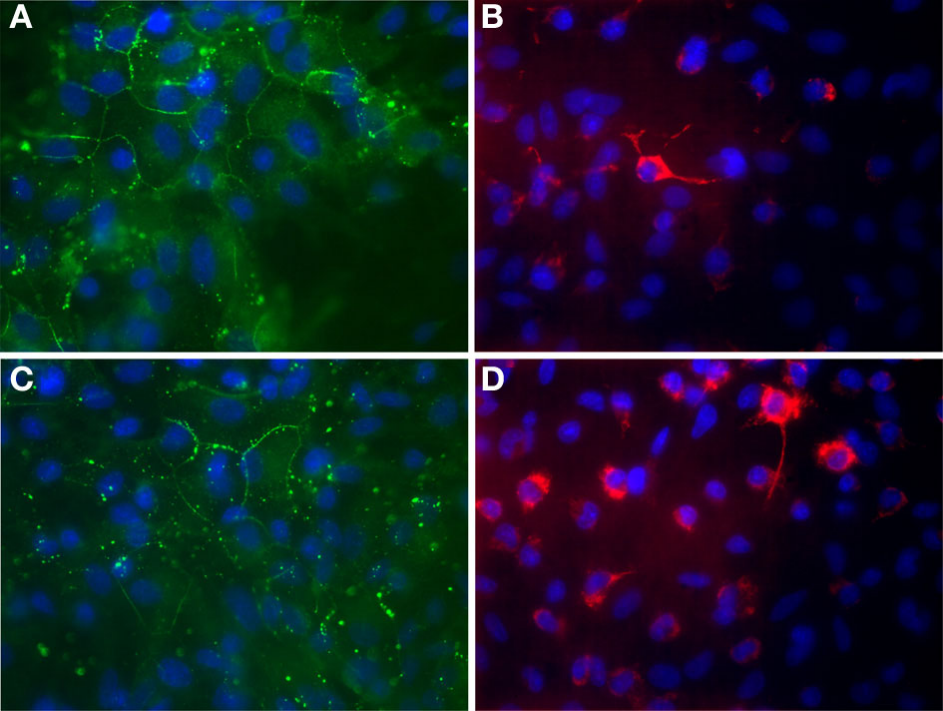
**Immunocytochemical labeling of Cx43 expression (A,C) and ED-1 positive cells (B,D) of astroglia/microglia co-cultures. (A,B)** Astrocytes co-cultured with about 5% microglial cells in order to mimic physiological brain tissue. **(C,D)** Astrocytes co-cultured with about 30% microglial cells in order to mimic inflammatory affected brain tissue. Glial cells were counterstained with DAPI to visualize the nuclei (blue). **(A)** Astroglial Cx43 Expression (green) under physiological mimicked *in vitro* condition. **(B)** Microglia (red) are mostly found as inactivated, resting ramified type under physiological condition. **(C)** Astroglial Cx43 Expression (green) under inflammatory mimicked *in vitro* condition. The Cx43 protein level is reduced in those cultures. **(D)** Microglia (red) proliferate and change their phenotype to a round activated form under inflammatory condition. This process could also be observed under *in vitro* conditions in cultures. 63x Magnification.

Hinkerohe et al. investigated the role of DEX on functional coupling and Cx43 expression of three different glioma cell lines. They found that DEX decreases both functional GJC and Cx43 protein expression in all three cell lines (Hinkerohe et al., 2011). They also used a co-culture model of astrocyte-microglia which had a different yield in microglia numbers and demonstrated that microglia play an important role in Cx43 expression of astrocytes (Figure 1) (Faustmann et al., 2003). In their *in vitro* model DEX had no effect on astrocyte-microglia cultures containing a low number of microglia (M5) in respect to functional coupling, membrane resting potential (MRP), Cx43 expression and microglia morphology. On the contrary, in cultures with a high number of microglia (M30), DEX increased Cx43 expression and GJC and decreased microglia activity based on morphology assessment. Hinkerohe et al. claimed that this pattern is an *in vitro* mimic of glioma in the brain, where M5 condition is representing a healthy tissue and M30 stands for pathologic conditions located in the close vicinity of glioma mass.

Similarly, the application of DEX in *in vitro* cultures of three different cell lines reduced the bystander effect of HSV-TK gene therapy, as well as GJC and sensitivity of tranfected cells to ganciclovir (Robe et al., 2005). Although the *in vitro* results should be carefully interpreted to be applied *in vivo*, they infer the point that DEX administration in glioma could have a negative impact on glioma treatment and should be handled cautiously.

#### Anti-epileptics, gap junctions, and glioma

Beside cytotoxic medication, patients commonly receive symptomatic therapies in cancer. AEDs are used to treat seizures, one of the most common complications in brain tumors (Van Breemen et al., 2007). In conjunction with their role in controlling seizure, they were also proposed to have a role in reducing tumor growth.

Sodium valproate (VPA) is commonly used as AED but it has another function as a histone deacetylase inhibitor (HDAC). HDACs have anti-cancer effects and can also modulate GJs in glioma cell lines (Ammerpohl et al., 2004; Asklund et al., 2004; Shao et al., 2004; Kuendgen and Gattermann, 2007; Duenas-Gonzalez et al., 2008). On the other hand, glioma cells express Cx26 in lower amounts, as well (Estin et al., 1999). Ryu et al. investigated VPA's role in HSV-TK gene therapy in U87 human glioma cells and showed that the expression of Cx43 and Cx26 was increased by VPA treatment (Ryu et al., 2012).

However, according to the meta-analysis study by Sirven et al., none of the three evaluated AEDs (phenobarbital, phenytoin, VPA) could indicate beneficial effects as seizure prophylaxis in glioma (Sirven et al., 2004) nor a correlation between VPA use and survival rate was reported (Van Breemen et al., 2009). On the contrary, in another study, AEDs, especially VPA, increased survival rates in glioma patients (Guthrie and Eljamel, 2013). The controversy in these findings could raise the question of whether the intrinsic characteristics (e.g., GJs expression) of tumor cells or the stage of tumor are responsible for the diversity of the reaction of AEDs in glioma treatment. In general, the remarkable aspect of VPA as being anti-cancer agent (HDAC) and AED (GJs modulations) makes it an interesting medication to investigate in glioma treatment.

#### Homocellular and heterocellular gap junctional coupling

All mentioned studies evaluated the role of GJs in homocellular population; nevertheless, we should also consider the role of heterojunctional coupling between astrocyte and glioma cells (Oliveira et al., 2005) in pharmacological studies. Both glioma and astrocytes express similar Cxs which hypothetically allow them to connect through membranes and transfer metabolites and certain molecules. The transfer of such molecules or metabolites can be either detrimental or beneficial to cell proliferations of both tumor cells and astrocytes. In addition, the other elements residing in the brain tissue can modulate other factors such as the blood flow of brain tissue. These changes can dramatically affect tumor growth and their micro-milieu. For example, during or after ischemia, the glucose metabolism can oppositely modulate the effect of GJs on neuronal survival (Farahani et al., 2005). These modifications can differentially affect GJC in astrocytes, tumor cells or the combination of both.

#### Conclusion

With respect to GJs, there is evidence that less integrity within glioma cell population and more integrity with the surrounding astrocytes would contribute to the migration of glioma cells (Sin et al., 2012). Therefore, it is important to find a GJ selective drug that is differentially affecting tumor cells and astrocytes. This means GJs modulator should act in favor of astrocyte survival and tumor cell eradication. Thus, far, Cx43 modulation did not show a clear advantage in glioma treatment. However, further experiments would clarify and probably introduce new treatments in the future. Lastly, studies on the role of anti-inflammatory, anti-cancer and AEDs on the co-cultures of astrocyte-glioma cells could provide more information on the therapeutic role of GJs in glioma.

### Epilepsy

#### Introduction

Epilepsy is one of the most common neurological disorders affecting about 1% of the world population. According to the definition of International League Against Epilepsy (ILAE) and International Bureau of Epilepsy (IBE), epilepsy is a disorder of the brain accompanied by neurologic, cognitive, psychological, and social consequences of continued predisposition in the brain that causes epileptic seizures (Fisher et al., 2005). Epilepsy can have various reasons: traumatic brain injury, genetic predisposition, stroke, or post-inflammatory responses in CNS. The main focus of therapeutics is on reducing uncontrollable neuronal firing in patients. Although neurons are thought to be the main cause of epilepsy, glial cells gradually receive more attention because of their direct interaction with neurons termed as neuronal-glial network. In this network, glial cells take part in the modulation of synaptic transmission through modifications in channels, transporters, and receptors as well as GJs (Binder and Steinhauser, 2006; Steinhauser et al., 2012; Binder and Carson, 2013). In the following, the influence of AEDs on GJs and their potential role on epilepsy will be discussed.

#### Anti-inflammatory drug and gap junctions

The blockade of GJs has been referred to reduced seizure activity in animal models. Investigating the anticonvulsant potential of GJ blockade, Nilsen et al. (2006) and Jin et al. (2013) applied meclofenamic acid (MFA) in epileptic rodent models. They showed that MFA reduces seizure by blocking neuronal Cx36 as well as astrocytic Cx43. The mechanism by which MFA caused this effect is unknown; however, the anti-inflammatory and strong GJs blockade properties of MFA could both play a role. MFA belongs to non-steroidal anti-inflammatory drugs (NSAID) family that inhibits cyclooxygenase (COX) pathways of phospholipid degradation. The final results of COX activation is prostaglandin (PG) synthesis and consequently, inflammation. Therefore, MFA, as NSAID, can affect the micro-milieu in which neurons, astrocytes and microglia reside and reduce the inflammation caused by phospholipid degradation. On the other hand, although GJs' functional activity is reduced by inflammation, MFA, as an anti-inflammatory drug, reduced GJC on astrocytes, as well. Whether MFA has direct or indirect (via COX inhibition, PG synthesis, and micro-milieu modification) effects on GJs activity in seizure will remain a question to be explored by further studies. Nevertheless, these results support the assumption of the proposed role of GJs in the seizures' generation and propagation. Considering the inflammatory theory for seizure (Vezzani and Granata, 2005; Vezzani et al., 2011, 2013), the role of anti-inflammatory cascades caused by anti-inflammatory drugs on GJs and epilepsy is worth to investigate.

#### Gap junctional blockade and epilepsy

Introducing GJs as possible interacting partners with neurons in synapses (Araque et al., 1999), the impression of reducing neuronal firing in epilepsy by manipulating GJs were examined. Therefore, Carbenoxolone (CBX) as a non-selective GJ blocker that exerts anti-epileptic effect in animal models was studied (Gigout et al., 2006). The CBX effect on neurons decreases the cumulative duration of cortical spike-wave discharges in an adult rat genetic model of absence epilepsy. CBX also diminished seizure-like primary after discharges in the rat CA1 hippocampal pyramidal region and increased neuronal excitability in whole-cell recordings (Jahromi et al., 2002). Beside neurons, CBX can also affect astrocytes. Volume-regulated anion channels (VRAC) are activated by hypotonic challenges in cultured rat cortical astrocytes and low concentrations of CBX could inhibit this effect. However, the same effect of CXB was observed in Cx43 Knockout astrocytes (Benfenati et al., 2009). These results could imply the point that CBX effect in epilepsy is probably mediated through mechanisms other than Cx43 inhibition. Nevertheless, controversial findings from other experimental studies require more delicate methods and interpretation of the effect of GJ blockade and epilepsy.

#### Anti-epileptic drugs and gap junctions

The effect of some common AEDs including phenytoin (PHE), carbamazepine (CBZ), gabapentin (GBT), and VPA on the astroglial Cx43 expression in astroglia/microglia cultures of newborn rats was recently investigated (Dambach et al., 2014). In this study, astrocytes were co-cultured with different percentages of microglial cells (M5 or M30). Incubation with different concentrations of these AEDs, based on the levels of AEDs in liquor of the patients, did not influence astroglial Cx43 expression. This study could not provide an obvious role for AEDs with regard to GJs modulation. The number of experiments and the nature of the study of being *in vitro* could mask the possible effect in this context.

However, in the study of Haghikia et al. levetiracetam (LEV) increased Cx43 expression and GJC in astrocytes and restored impaired astrocyte MRP via modification of inward and outward rectifier currents in cultures with higher counts of microglia (Haghikia et al., 2008; Stienen et al., 2011). The transfer of a fluorescent dye from injected cells to the surrounding ones, was considered as an indicator of GJC (in this experiment Cx43) activity. Participation of astrocytes in neural synapses as excitable cells has not been completely confirmed. Nevertheless, due to the connection of astrocytes and neurons via GJs, LEV can be a potential modulator of neuronal excitability, as well.

#### Conclusion

Recent studies showed a role for inflammation and anti-inflammatory drugs in epilepsy. Besides, microglia as a prominent functional cell in inflammation has gained especial attention in epilepsy. Likewise, modification of astrocytic Cx43 by microglia has been investigated by several groups. Based on these findings, manipulation of microglia to reduce inflammation would be beneficial in epilepsy treatment. For example, a decrease in GJ permeability can oppositely affect neuronal excitability by reflecting two aspects: (1) a fast, pro-convulsive effect due to impaired K^+^ redistribution, (2) delayed anti-epileptic effect because of disruption of neuronal energy supply, which is mediated through astrocytes (Seifert et al., 2006, 2010). Whether the final goal should be reducing or increasing Cx43 is still an open question. However, modulation of GJs in epilepsy remains a potential tool in epilepsy treatment.

## Outlook

In conclusion, the *in vitro* pharmacological studies on astrocytic GJs are sparse but have potential promising outcome for the treatment of different brain diseases, especially glioma and epilepsy. Table 1 summarizes the current information on the drug effects and clinical applications of GJs in brain illnesses. Although GJ manipulations do not function as a sole factor in treatment of brain diseases, it can serve as a predicting factor in the prognosis of specific therapeutics as well as a contributing factor in the etiology of certain CNS illnesses. Further studies on this topic are warranted to signify GJs modulations under pharmacological treatment.

**Table 1:** **Summary of the available information in regard to GJs and brain pathologies in *in vitro* studies**.

**Disorder**	**Drug**	**Connexin**	**Tissue**	**Cell type**	**Effect**	**References**
Migraine with aura	SB-220453 (Tonabersat)	Cx26	Trigeminal	Satellite ganglial cell, neuron	↓ expression of Cx26	Damodaram et al., 2009
			Nerve		↓ CSD	
					↓ migraine attacks	
Multiple sclerosis	FTY720 (Fingolimod)	Cx43	Brain	Astrocyte	↓ of GJC	Rouach et al., 2006
					↑ dephosphorylated Cx43	
Multiple sclerosis	IFNβ (Interferon-β)	Cx43	Brain	Astrocyte	Restored astrocyte depolarization restored	Hinkerohe et al., 2005
					↓ GJC	
Glioma	Dexamethasone	CX43	Brain	Glioma cell line	↓ GJC	Hinkerohe et al., 2011
					↓ Cx43 expression	
Glioma	Sodium valproate	Cx43,Cx26	Brain	Glioma cell line	↑ Cx43,Cx26 expression	Ryu et al., 2012
Brain inflammation	Dexamethasone	Cx43	Brain	Astrocyte	↑ GJC	Hinkerohe et al., 2005
					↑ Cx43 expression	
Epilepsy	AEDs (Phenytoin, Gabapentin, Sodium valproate, Carbamazepine, Levetiracetam)	Cx43	Brain	Astrocyte	↑ GJC and ↑ Cx43 expression by Levetiracetam, no change on others	Haghikia et al., 2008; Dambach et al., 2014

*Cx, connexin; GJC, gap junctional communication; CSD, cortical spreading depression*.

### Conflict of interest statement

The authors declare that the research was conducted in the absence of any commercial or financial relationships that could be construed as a potential conflict of interest.
